# Supplementary material for: Causal Relationship between Aging and Anorexia Nervosa: A White-Matter-Microstructure-Mediated Mendelian Randomization Analysis
Source: Biomedicines. 2024 Aug 16;12(8):1874. doi: 10.3390/biomedicines12081874 (PMC11351342; doi:10.3390/biomedicines12081874)
Supplement: Supplementary file 1 [file biomedicines-12-01874-s001.zip › Supplementary Figure/Figure S9/Leaveoneout plot of MR analysis for ubm-b-1896 to AN.pdf]

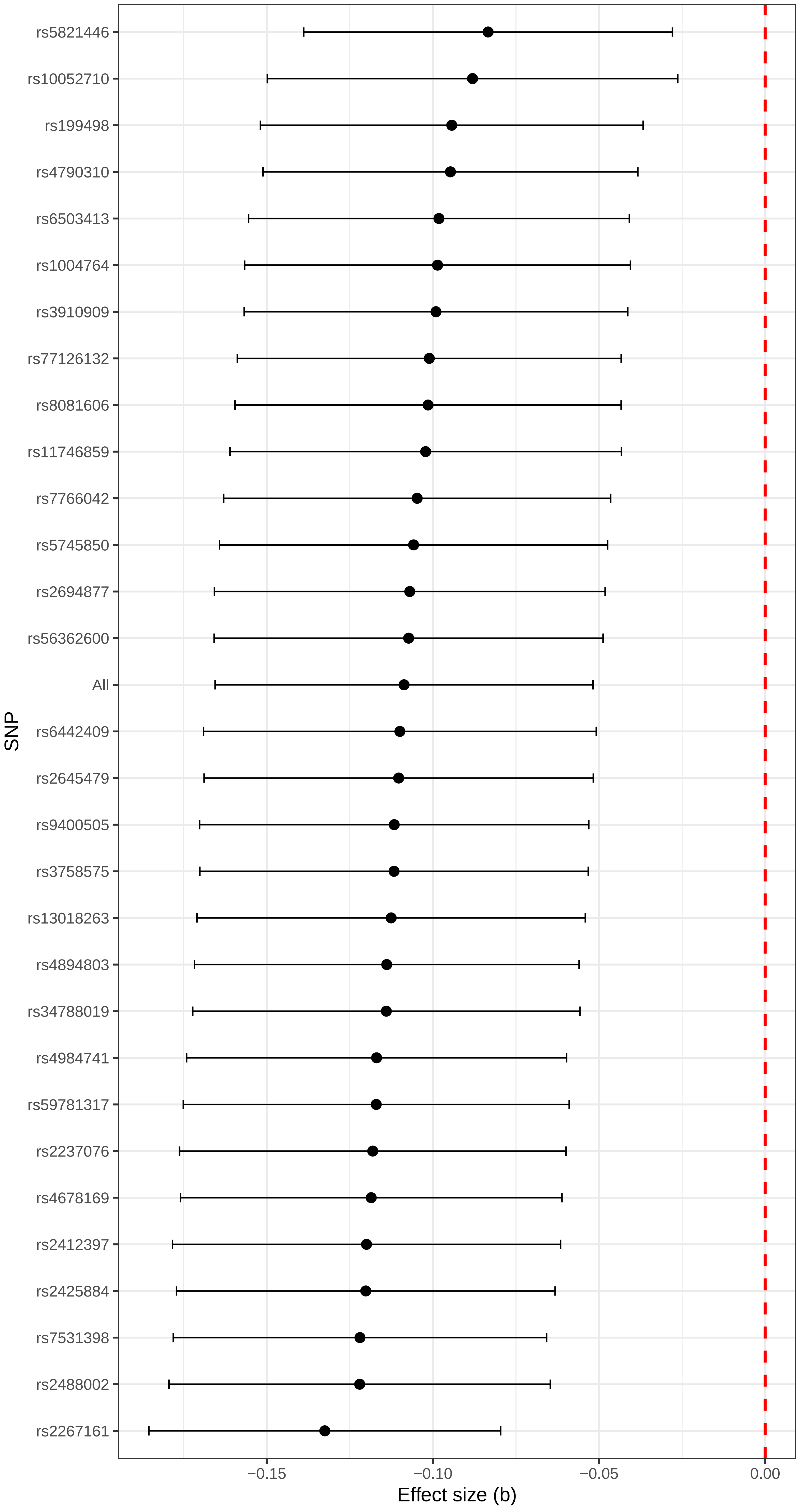

rs5821446
rs10052710
rs199498
rs4790310
rs6503413
rs1004764
rs3910909
rs77126132
rs8081606
rs11746859
rs7766042
rs5745850
rs2694877
rs56362600
All
rs6442409
rs2645479
rs9400505
rs3758575
rs13018263
rs4894803
rs34788019
rs4984741
rs59781317
rs2237076
rs4678169
rs2412397
rs2425884
rs7531398
rs2488002
rs2267161
SNP
−0.15
−0.10
−0.05
0.00
Effect size (b)
